# Impella RP for Patients with Acute Right Ventricular Failure and Cardiogenic Shock: A Subanalysis from the IMP-IT Registry

**DOI:** 10.3390/jpm12091481

**Published:** 2022-09-09

**Authors:** Giulia Botti, Mario Gramegna, Francesco Burzotta, Giulia Masiero, Carlo Briguori, Carlo Trani, Massimo Napodano, Anna Mara Scandroglio, Matteo Montorfano, Giuseppe Tarantini, Alaide Chieffo

**Affiliations:** 1 Vita Salute San Raffaele University, 20132 Milan, Italy; 2 Interventional Cardiology Unit, IRCCS San Raffaele Scientific Institute, 20132 Milan, Italy; 3 Cardiac and Cardiac Surgery Intensive Care Unit, IRCCS San Raffaele Scientific Institute, 20132 Milan, Italy; 4 Department of Cardiovascular Sciences, Fondazione Policlinico Universitario A. Gemelli IRCCS, Università Cattolica del Sacro Cuore, 00168 Rome, Italy; 5 Department of Cardiac, Thoracic, Vascular Sciences, and Public Health, University of Padua Medical School, 35128 Padua, Italy; 6 Laboratory of Interventional Cardiology and Department of Cardiology, Mediterranea Cardiocentro, 80122 Naples, Italy

**Keywords:** cardiogenic shock, Impella RP, percutaneous right ventricular assist device, right ventricular failure

## Abstract

The use of percutaneous right ventricular assist devices (pRVADs) to support patients with right ventricular (RV)-predominant cardiogenic shock (CS) refractory to optimal medical therapy is increasing progressively, and the Impella RP is the first FDA-approved pRVAD in such a clinical scenario. The aim of the present study is to report the outcomes of patients treated with Impella RP in the IMP-IT (IMPella Mechanical Circulatory Support Device in Italy) registry, a multicenter registry that evaluated the trends in use and clinical outcomes of the Impella in the setting of CS and high-risk percutaneous coronary intervention in Italy. A total of 15 patients who received Impella RP were enrolled. In 40% of the patients, the main cause was ST-segment elevation myocardial infarction. A total of 40% of patients required biventricular support with a left Impella. Device-related complications were reported in 46.7% of patients. Overall, the in-hospital mortality was 46.7%, whereas the one-year mortality was 53.3%. The composite rate of all-cause death, heart failure (HF) hospitalization, left ventricular assist device (LVAD) and heart transplant at one year was 60%. The Impella RP has favorable survival outcomes in RV-predominant cardiogenic shock. However, the device-related complications are frequent and should be carefully weighed when considering escalation to Impella RP.

## 1. Introduction

Overall, the use of percutaneous right ventricular assist devices (pRVADs) may be a therapeutic option to support patients presenting with right ventricular (RV)-predominant cardiogenic shock [1,2]. Such a clinical scenario is characterized by a sudden alteration of the right ventricular loading, resulting in a precipitation of the function of the right chamber [2]. Multiple mechanisms have been suggested to be at the origin of the RV-predominant cardiogenic shock; however, consensus on the definition and optimal strategies for diagnosis is still a matter of debate [2,3]. Commonly, clinical and echocardiographic parameters are the first indicators of cardiogenic shock and right ventricular dysfunction. Nevertheless, an invasive hemodynamic assessment with pulmonary artery catheterization is still considered valuable both diagnostically and prognostically [4]. The rationale for the use of pRVADs is to allow for the rapid stabilization of patients that are refractory to optimal medical therapy, protecting the myocardial tissue from ischemia and promoting its morphological and functional recovery [1,3]. The Impella RP (Abiomed, Danvers, MA, USA), a microaxial flow pump, is the first FDA-approved pRVAD for patients with medically refractory RV failure. The device is a percutaneous, catheter-based pump that is inserted via a single transfemoral venous access; it is composed of an inflow section placed in the right atrium, and an outflow section is positioned in the pulmonary artery to allow for bypass of the right ventricle. The Impella RP provides circulatory support by ensuring stable blood flow through the right chambers, as well as protecting the ventricle from the threat of increased afterload. It has shown promising outcomes in early studies [5,6]. Although the experience is limited when compared to the use of the Impella devices for left ventricular support [7,8,9], a small number of studies have shown that the Impella RP is effective and safe when used to treat refractory RV failure. The IMP-IT registry (IMPella Mechanical Circulatory Support Device in Italy) is an investigator-initiated, nationwide, all-comer, multicenter registry that evaluated the trends in use and clinical outcomes of the Impella in the setting of cardiogenic shock (CS) and high-risk percutaneous coronary intervention (HR-PCI) in Italy [10]. The aim of the present study is to report the outcomes of patients treated with Impella RP in the IMP-IT registry.

## 2. Materials and Methods

### 2.1. Study Population

The IMP-IT study is a retrospective observational national registry, promoted by the Italian Society of Interventional Cardiology (Società Italiana di Cardiologia Interventistica—GISE), including all consecutive patients treated with Impella 2.5, Impella CP, Impella 5.0 and Impella RP, both for CS and HR-PCI, in 17 Italian centers from 2004 to June 2018. The study design, including the centers and collection of records, has been described elsewhere [10]. Data related to medical history, procedural characteristics and in-hospital and one-year outcomes were collected from each center and included in a pre-specified structured dataset. Clinical follow-up data were collected by in-person visits, telephone interviews and medical notes from any hospital admission or outpatient visits. The adverse events were then adjudicated by two independent cardiologists using source documents provided by each center. A PCI was performed according to each center’s standard clinical practice. The collection of data at each participating site was performed according to the policies of the local institutional review board or ethics committee. For this study, all patients from the IMP-IT registry who received mechanical circulatory support with Impella RP were included, and the analysis was performed in the setting of CS only, as no patients were treated with Impella RP whilst undergoing HR-PCI. The indication for the implantation of the device was overt cardiogenic shock and not responding to optimal medical therapy according to hemodynamic and echocardiographic parameters. The description of the Impella RP device is available in Appendix A.

The subanalysis of patients treated with Impella RP was performed on retrospectively collected and completely anonymized data.

The objectives of this subanalysis were as follows: (1) to analyze the characteristics of the population receiving support with Impella RP; (2) to evaluate the in-hospital and one-year clinical outcomes in patients treated with Impella RP. The primary clinical endpoint was, as in the main analysis, the composite of death, rehospitalization for heart failure (HF), LVAD implantation or heart transplantation. The study endpoints and definitions are described in Appendix B.

### 2.2. Statistical Analysis

The patient data were selected from the IMP-IT dataset. The baseline characteristics are reported as a number (%), mean ± standard deviation or median (interquartile range). The event rates at the one-year follow-up were estimated using the Kaplan–Meier method as a time-to-first event. Due to the low number of events in the cohort, a predictive analysis was not performable. A statistical analysis was performed using XLSTAT version 24.3.1 (Addinsoft, Paris, France).

## 3. Results

### 3.1. Patient Population and Baseline Characteristics

A total of 15 patients who received Impella RP were enrolled in the analysis. The baseline characteristics and clinical presentation are reported in Table 1. The mean age was 67.8 ± 14.4 years old, 66.7% were male, and 20% and 13.3%, respectively, suffered from diabetes mellitus and chronic heart failure (HF). At the time of the index event, all patients presented with CS; the main cause being acute myocardial infarction (AMI) with ST-segment elevation (40.0%). Most of the patients had concomitant left ventricular dysfunction (EF 32.9 ± 13.7%). Two patients presented with out-of-hospital cardiac arrest. A total of 86.7% of patients required mechanical ventilation, whereas 46.7% were pharmacologically supported with inotropes.

The mean arterial pressure before the Impella RP insertion was 65.2 mmHg; the mean serum lactate was 5.1 mmol/L; the mean serum creatinine was 2.1 mg/dL.

### 3.2. Procedural Characteristics

The procedural characteristics are summarized in Table 2. Most of the patients (60%) received Impella RP only, whereas the remaining patients received biventricular support with Impella RP plus Impella 2.5 (13.3%) or Impella RP plus Impella CP (26.7%). Additional mechanical support with an intra-aortic balloon pump (IABP) was required in 46.7% of patients. The mean duration of the Impella RP support was 156.0 ± 92.1 h, and the intensive care length of the stay was 15 days. Coronary angiography was performed in 53.3% of patients, and 33.3% underwent PCI. Of the eight patients undergoing PCI, six were admitted with STEMI. The angiographic and procedural characteristics are reported in Table 3.

### 3.3. In-Hospital Outcomes

The in-hospital outcomes are reported in Table 4. The rate of the overall in-hospital mortality was 46.7%. Hemolysis and limb ischemia were reported in 26.6% and 20% of patients, respectively. An endovascular intervention was needed in all of the latter patients (20%). A total of 66.7% of patients suffered acute kidney injury, and 46.7% required renal replacement therapy. Two patients required escalation therapy and, more specifically, one received support with extracorporeal membrane oxygenation (ECMO), while the other underwent an implantation of a left ventricular assist device (LVAD). Regarding the concomitant left ventricular dysfunction, the mean ejection fraction upon discharge from the hospital was 38.5%. In our study, the rate of sepsis is quite high (60%); however, the development of this condition was secondary to an infection of the Impella implantation site in one case only. On the other hand, all patients who developed sepsis, apart from the aforementioned, underwent mechanical ventilation and were treated in an intensive care unit, which is known to be an infection-predisposing environment.

### 3.4. One-Year Outcomes

**Figure 1:**
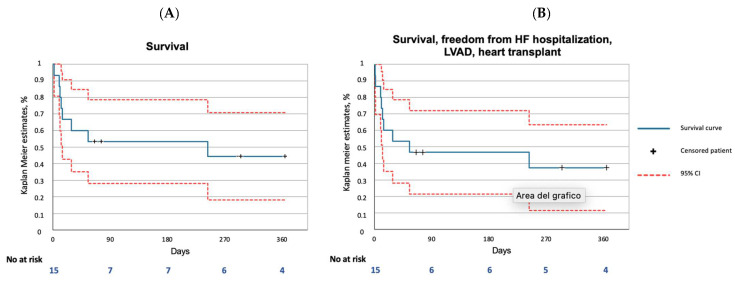
One-year outcomes in the Impella RP subanalysis of the IMP-IT registry. (**A**) Survival; (**B**) Composite of survival, freedom from HF hospitalization, LVAD and heart transplantation. HF, heart failure; LVAD, left ventricular assist device; CI, confidence interval.

The one-year outcomes are reported in Table 5 and in Figure 1A,B. The all-cause death rate was 53.3%, and cardiac death was the only cause. Further treatment with LVAD was necessary in one case. The composite rate of all-cause death, HF hospitalization, LVAD and heart transplant was 60%.

## 4. Discussion

The main findings of this substudy from the IMP-IT registry are the following: (1) The in-hospital mortality rate of patients treated with Impella RP in the setting of CS is significant; however, the one-year mortality rate minimally differed from the latter, which may imply that patients who are safely discharged from hospital make a full recovery in this perspective. (2) The rate of escalation to VAD or transplant is extremely low, which, again, may suggest that the right ventricle is capable of a complete and durable structural and functional recovery; interestingly, no cases of one-year heart failure rehospitalization were reported, but this might be due to the limited number of patients. (3) The rate of complications is quite significant (46.7%); therefore, escalation to Impella RP should be carefully weighed in such severely compromised patients.

RV-predominant cardiogenic shock is associated with poor morbidity and mortality. However, the RV has been demonstrated to be capable of a rapid and complete recovery and more resilient to ischemic insult when compared to the left-sided heart [11]. From this perspective, the choice of percutaneous, temporary, mechanical support can become a potentially attractive option [5,12]. According to the 2021 ESC guidelines for the diagnosis and treatment of acute and chronic heart failure, MCS in the setting of isolated RVF and CS should be considered as a second line of treatment for patients who do not respond to medical treatment with vasopressors or inotropes (Class IIa, C) [13].

Currently, there are no randomized controlled trials for the use of Impella RP, and systematical, registry-derived data is limited to the RECOVER RIGHT registry, which includes 30 patients treated with Impella RP, and to its most recent update, in which the cohort has been expanded to 60 patients [5,6]. When comparing the outcomes of the latter studies with the ones registered in this subanalysis, one might notice a remarkable difference in the overall mortality rates (26.7% vs. 53.3%). However, the RECOVER RIGHT registry is composed of the following two cohorts of patients: Cohort A, treated with Impella RP after LVAD implantation, and Cohort B, treated in the setting of AMI-CS, post-cardiotomy and post-transplant. The mortality rates of the RECOVER RIGHT registry Cohort B and of the IMP-IT Impella RP substudy are similar (41.1% vs. 53.3%). Therefore, our results are in line with previously published data.

To date, the numerous published case reports and case series underline the wide variety of clinical scenarios in which treatment with Impella RP might be beneficial in stabilizing the patient’s hemodynamics. These span from RV failure following LVAD implantation, AMICS, acute myocarditis, acute pulmonary embolism and CS following unsuccessful right coronary revascularization [14,15,16,17,18]. Most papers convey concordant results regarding hemodynamic improvements and favorable survival rates. However, the morbidity burden remains notably high with significant rates of hemolysis, severe bleeding and acute limb ischemia. In this perspective, randomized trials comparing the escalation to Impella RP with medical therapy might pave the way for guideline indications for the use and timing of Impella RP, as well as somehow justify the rate of complications. Currently, this comparison is limited to studies on animal models [19].

Of particular importance is the understanding of the concomitant degree of LV failure in patients presenting with acute RVF. In our cohort, the mean ejection fraction (EF) was 32.9%, and a considerable number of patients required some kind of biventricular support. For a patient with biventricular failure, an isolated pRVAD risks further decompensation of left ventricular failure, obligating concurrent consideration of pharmacologic and mechanical options for left-sided support. Therefore, the investigation and standardization of the optimal timing and sequencing of the treatment might lead to optimization of the outcomes.

Our study has several limitations that need to be disclosed. Firstly, the limited number of enrolled patients is a limitation to making certain conclusions. The analyses are performed on retrospectively collected data, therefore, in the absence of event monitoring and of standardized diagnostic and therapeutic criteria. However, the concordance of our outcomes with those of previously published studies strengthens the reliability of our results.

## 5. Conclusions

The implementation of pRVADs, or, more specifically, of Impella RP, in the treatment scheme for patients suffering from acute RVF refractory to medical therapy is feasible in terms of survival outcomes; however, the morbidity burden is not negligible and should be considered when escalating to Impella RP. Moreover, most patients present with biventricular failure and require a combination of treatments to support both ventricles. Our results, along with other available published data, strongly highlight the need for prospective research, powered to support the formulation of guidelines for timing and escalation to Impella RP.

## Figures and Tables

**Table 1:** Baseline characteristics and clinical presentation.

	Patients (n = 15)
Age	67.8 ± 14.4
Male	10 (66.7)
Hypertension	8 (53.3)
Dyslipidaemia	5 (33.3)
Diabetes mellitus	3 (20.0)
Chronic pulmonary disease	4 (26.7)
Prior myocardial infarction	3 (20.0)
Previous percutaneous coronary intervention	5 (33.3)
Previous coronary artery bypass graft	-
Chronic kidney disease *	4 (26.7)
Dialysis	2 (13.3)
Atrial fibrillation	4 (26.7)
Prior transient ischaemic attack or stroke	2 (13.3)
Peripheral artery disease	1 (6.7)
Chronic heart failure	2 (13.3)
Left ventricular ejection fraction, %	32.9 ± 13.7
Right ventricular dysfunction	15 (100)
Out-of-hospital cardiac arrest	2 (13.3)
Aetiology of cardiogenic shock	
ST-elevation myocardial infarction	6 (40.0)
Acute myocarditis	2 (13.3)
Other	7 (46.7)
Haemodynamic Values	
Heart rate, bpm	85.9 ± 25.0
Mean arterial pressure, mmHg	65.2 ± 11.2
Laboratory values	
pH	7.34 ± 0.1
Serum lactate, mmol/L	5.1 ± 3.9
Haemoglobin, g/dL	12.2 ± 2.0
Serum creatinine, mg/dL	2.1 ± 1.2

Results are reported as n (%) and mean ± standard deviation as appropriate. * Defined as eGFR < 60 mL/min/1.73 m^2^. One patient suffered from RVF after transapical TAVI; one patient suffered from RVF after mitral valve replacement surgery.

**Table 2:** Clinical course and procedural characteristics.

Other Concomitant pVAD	Patients (n = 15)
Isolated Impella RP	9 (60.0)
Impella RP + left-side Impella (BiPELLA)	6 (40.0)
Impella 2.5	2 (13.3)
Impella CP	4 (26.7)
Impella 5.0	-
Intra-aortic balloon pump	7 (46.7)
Inotropes	7 (46.7)
Mechanical ventilation	13 (86.7)
Duration of Impella support, hours	156.0 ± 92.1
Length of mechanical ventilation, hours	96 (48–252)
Intensive care length of stay, days	15 (10–27)

Results are reported as n (%) for the categorical variables and as a median (interquartile range) or mean ± standard deviation for the continuous variables as appropriate.

**Table 3:** Angiographic and procedural characteristics.

	Patients (n = 8)
Coronary angiography performed	8 (53.3)
PCI performed	5 (33.3)
Left main disease	1 (6.7)
Left anterior descending artery disease	3 (20.0)
Left circumflex disease	3 (20.0)
Right coronary artery disease	6 (40.0)
Number of diseased vessels	1.1 ± 1.2
Three-vessel disease	2 (13.3)

Results are reported as n (%) for the categorical variables and as a median (interquartile range) or mean ± standard deviation for the continuous variables as appropriate; PCI, percutaneous coronary intervention.

**Table 4:** In-hospital outcomes.

	Patients (n = 15)
Death	7 (46.7)
Life-threatening or severe bleeding	-
Number of red blood cell transfusions	8.79 ± 9.6
Device-related complications	
Access-site bleeding	-
Haemolysis	4 (26.6)
Limb ischaemia	3 (20.0)
Sepsis	9 (60.0)
Acute kidney injury *	10 (66.7)
Need for renal replacement therapy	7 (46.7)
Escalation therapy	2 (13.3)
LVEF at discharge, %	35.8 ± 17.7

In-hospital outcomes are reported as n (%) or mean ± standard deviation as appropriate; LVEF, left ventricular ejection fraction. * Defined as a serum creatinine increase ≥ 0.3 mg/dL from the baseline. Defined as the need for extracorporeal membrane oxygenation, left ventricular assist device implantation or heart transplant.

**Table 5:** One-year outcomes.

	Patients (n = 15)
All-cause death	8 (53.33)
Cardiac death	8 (53.3)
Hospitalisation for heart failure	-
Myocardial infarction	-
Stroke	2 (13.3)
LVAD or heart transplant	1 (6.7)
Death, hospitalization for heart failure, LVAD or heart transplant	9 (60.0)

One-year outcomes are reported as a number of events (%).

## Data Availability

The data supporting the results presented in this research are available upon reasonable request.

## Appendix A. Description of the Impella RP Device

The Impella RP is a percutaneous, catheter-based, microaxial flow pump that allows an output of up to 4 L/min. The Impella RP is composed of a 22Fr impeller mounted onto an 11Fr catheter with the inflow positioned at the level of the inferior vena cava and the outflow into the pulmonary artery, allowing for a right ventricle bypass. The device is inserted via a 23Fr venous peel-away sheath through a single transfemoral (usually right) venous access.

## Appendix B. Study Endpoints and Definitions

*In-hospital death* was defined as any patient who died during the hospital stay.

*Need for renal replacement therapy* (*RRT*) is the utilization of any modality of RRT in the case of little or no residual kidney function.

*Acute kidney injury* was defined as any of the following: an increase in the serum creatinine by ≥0.3 mg/dL (≥26.5 lmol/L) within 48 h; an increase in the serum creatinine to ≥1.5 times from the baseline, which is known or presumed to have occurred within the prior seven days; or a urine volume <0.5 mL/kg/hr for six hours.

*Need for mechanical ventilation* was defined as the need for invasive ventilatory support by endotracheal tube placement.

*Need for support escalation due to hemodynamic deterioration* was defined as left or right ventricular failure that is not responsive to Impella support and requires the use of advanced short-term mechanical support, such as ECMO, or the need for, in patients dependent on mechanical support, transplantation or long-term mechanical support, such as surgical implantation of LVAD.

*Need for LVAD/transplantation* was defined as cardiac transplantation or long-term mechanical circulatory support (LVAD) in patients in INTERMACS class I, II or III during hospital stay or in patients in INTERMACS class IV after discharge.

*Device-related complications* were defined as vascular access complications in terms of bleeding or limb ischemia, vascular complications requiring endovascular interventions, neurological events (stroke), life-threatening bleeding, hemolysis, number of RBC transfused after Impella insertion and aortic injury, such as dissection or left ventricular perforation.

*Neurological events* were defined as follows:

− Stroke is defined as the duration of a focal or global neurological deficit ≥24 h; or <24 h if the available neuroimaging documents a new hemorrhage or infarct or if the neurological deficit results in death.
− Transient ischemic attack is defined as the duration of a focal or global neurological deficit <24 h; any variable neuroimaging does not demonstrate a new hemorrhage or infarct.

*Bleeding* was defined according to the Global Use of Strategies to Open Occluded Arteries (GUSTO) criteria as follows: (i) severe or life-threatening: intracerebral hemorrhage or bleeding resulting in substantial hemodynamic compromise that requires treatment; (ii) moderate: requiring blood transfusion but not resulting in hemodynamic compromise; (iii) mild: bleeding that does not meet the above criteria.

*Hemolysis* was defined according to the INTERMACS definitions as follows: (i) major hemolysis: a plasma-free hemoglobin value greater than 20 mg/dL or a serum lactate dehydrogenase (LDH) level greater than two and one-half times (2.5×) of the upper limits of the normal range at the implanting center occurring after the first 72 h of a post implant and associated with clinical symptoms or findings of hemolysis or abnormal pump function. Major hemolysis requires the presence of one or more of the following conditions: hemoglobinuria (“tea-colored urine”); anemia (decrease in hematocrit or hemoglobin levels that is out of proportion to the levels explainable by chronic illness or usual post-VAD states); hyperbilirubinemia (total bilirubin is above 2 mg% with a predominately indirect component); pump malfunction and/or abnormal pump parameters; (ii) minor hemolysis: a plasma-free hemoglobin value greater than 20 mg/dL or a serum lactate dehydrogenase (LDH) level greater than two and one-half times (2.5×) of the upper limits of the normal range at the implanting center occurring after the first 72 h of a post implant in the absence of clinical symptoms or findings of hemolysis or abnormal pump function.
